# Porous biochars derived from brewery waste for the treatment of Cr(VI)-contaminated water

**DOI:** 10.1371/journal.pone.0314522

**Published:** 2024-11-26

**Authors:** Zeleke Zewde, Tsegaye Girma Asere, Menberu Yitbarek

**Affiliations:** Department of Chemistry, College of Natural Sciences, Jimma University, Jimma, Ethiopia; University of South Africa, SOUTH AFRICA

## Abstract

The use of brewery waste for the removal of pollutants such as chromium has rarely been studied. In the present work, the removal of hexavalent chromium (Cr(VI)) from aqueous solutions was evaluated by brewer’s spent grain (BSG), brewing sewage sludge (BSS), and their mixture (MIX), which were obtained from the Bedele Brewery Share Company, Ethiopia. BSG with acid and heat treatment at 600 °C was selected during the preliminary screening experiments and further characterized via FTIR, XRD, and SEM. An adsorption experiment was carried out in batches to study the effectiveness of adsorbents in removing Cr(VI) under different conditions. Factors affecting adsorption, including pH, contact time, adsorbent dosage, and initial Cr(VI) concentration, were analyzed and optimized. The best conditions for the highest efficiency in removing Cr(VI) were a contact time of 7 h, initial solution pH of 2, initial Cr(VI) concentration of 40 mg/L, and adsorbent dose of 2 g/L. The pseudo-second-order (PSO) model, which suggests chemisorption of Cr(VI) on the surface of the adsorbent, describes the kinetics of Cr(VI) removal by the adsorbent (R^2^ = 0.9570). The Freundlich isotherm was a good fit for the experimental equilibrium adsorption data. The BSG biochar was found to have an approximate adsorption capacity of 31.87 mg/g for Cr(VI). The ability to recycle adsorbents suggests that BSG biochar could be effectively used to treat Cr(VI) in wastewater. As a result, converting industrial waste into useful material is cost effective and beneficial for the protection of the environment. More research is recommended to study how well this adsorbent works in real wastewater samples and during the column adsorption process.

## 1. Introduction

The human environment has been negatively impacted by rapid population growth, urbanization, and industrial development due to pollution from waste and biodiversity loss. Industrialization, similar to other processes, has consequences for the environment, commonly leading to pollution and deterioration. This results in unavoidable expenses and effects related to the contamination of air and water sources, as well as the overall deterioration of the environment. Industrial waste is now the primary source of water pollution, and its annual growth is due to the industrialization of most countries [1].

High levels of pollutants are released into the environment through both natural and human activities, posing significant harm to human health and other living beings even at low levels, due to the lack of efficient removal methods [2, 3]. Heavy metals have a high density and are harmful even in small amounts [4]. Chromium, a heavy metal, is the 7^th^ most common element and can be found in trivalent chromium (Cr(III)) and hexavalent chromium (Cr(VI)) forms that enter water from industrial processes such as metal smelting, electroplating, tanning, metallurgy, and dyestuff. Cr(VI) is considered more dangerous than Cr(III) because of its greater water solubility, mobility, penetrability, oxidation, and carcinogenic potential [5]. Prolonged exposure to Cr(VI) in industrial settings may result in severe health conditions, including allergic skin reactions, skin sores, and lung tumors [6]. Hence, to reduce the potential health risk to humans and other organisms in the environment, it is necessary to treat wastewater containing harmful heavy metals such as Cr(VI) before it is released into nearby water sources via various treatment methods. Numerous methods, including electrochemical techniques, phytoremediation, chemical reduction, precipitation, membrane technology, photocatalytic reduction, ion exchange, and adsorption, have been studied for the removal of Cr(VI) [6]. Among those techniques, adsorption is considered a promising and commonly used method because of its selectivity, affordability, user-friendliness, effectiveness, and potential for material recycling [7].

Biomaterials have recently become more popular because of their high availability, low cost, and scalable and regenerative qualities. Different types of biomasses, including industrial wastes, agricultural wastes, and natural residues, are used as biosorbents for heavy metal remediation [8]. The beer industry generates large quantities of brewers’ spent grain (BSG) and brewery sewage sludge (BSS), creating environmental concerns that need sustainable management. The use of BSG and BSs as adsorbents can increase their value in addition to environmental management. BSG makes up approximately 85% of the overall byproducts produced during the brewing process. Typically, approximately 20 kg of BSG is generated per 100 L of beer made following the fermentation stage [9–11]. Worldwide, approximately 36.4 million tons of BSG are generated each year, making it a significant waste product that is commonly disposed of in landfills or used as animal fodder. This method of disposal is unsustainable since every ton of BSG in landfills has the potential to emit approximately 513 kg of CO_2_-equivalent greenhouse gases [12]. On the other hand, BSS is waste from wastewater plants at different stages of the beer production process. BSS is present in greater quantities than other byproducts generated during wastewater treatment. Sludge treatment requires additional facilities that may increase the overall treatment costs [13]. Therefore, an alternative approach for sludge management may involve the conversion of BSS into biochar for various applications, such as the adsorption of heavy metals from aqueous solutions.

Different techniques for the production of biochar from BSG, such as the in situ nitrogenation method [14], acrylic acid-coacryl amide modifying agent [15], base activation [16], zinc chloride activating agent [10] and hydrochloric acid modifying agent [17], have been used for the remediation of Cr(VI)-contaminated water. To the best of our knowledge, there is no comparative study on the application of biochars derived from BSG, BSS, or their mixture for the treatment of Cr(VI)-contaminated water. Therefore, this study investigated the adsorptive removal of Cr(VI) from aqueous solutions via biochars derived from phosphoric acid-treated brewery waste.

## 2. Materials and methods

### 2.1. Chemicals and reagents

The chemical used for this study was acetone (C_3_H_6_O, 99%, Sisco Research Laboratories Pvt. Ltd. India), 1,5-diphenyl carbazide (C_13_H_14_N_4_O, 98%, Analar, England), sulfuric acid (H_2_SO_4_, 98%, UNI CHEM, Germany), phosphoric acid (H_3_PO_4_, 98%, Nice Laboratories, India), nitric acid (HNO_3_, 69%, Loba Chemie Pvt. Ltd., India), hydrochloric acid (HCl, 35.4%; Loba Chemie Pvt. Ltd. India), potassium nitrate (KNO_3_, 99% Nice Laboratories), ammonia sulfate (NH_4_)_2_SO_4_ (99%, Riedel-Dehaën, Switzerland), disodium hydrogen phosphate (Na_2_HPO_4_, 99%, G-Biosciences, USA), sodium chloride (NaCl, 99.9%, Fisher Chemical, US) and sodium hydroxide (NaOH, 97%, Loba Chemie Pvt. Ltd. India), potassium dichromate (K_2_Cr_2_O_7_, 99.5%, LABMERK CHEMICALS PVT. LTD. All chemicals used in this study were of analytical reagent grade and were used without further purification.

### 2.2. Apparatus and instruments

An electronic balance (Model: JA103P, China), a furnace (Model: SX-4-10, Drawell, China), a water bath shaker (Model: GrantGLS400, England), an oven (Model: GENLAB WLDNES, England), a pH meter (Model: Bante902P, USA), a double-beam UV–Vis spectrophotometer (Model: SPECORD 200/PLUS, AnalytiK Jena, Germany), FT-IR (Model: PerkinElmer, Spectrum Two, USA), XRD (Model: Drawell XRD-7000) and SEM (Model: JCM-6000plus) were used. For recording the FTIR spectra, the sample was encapsulated in KBr via a hydraulic pellet press, and the spectra were taken in the wavenumber range of 400 cm^-1^ to 4000 cm^-1^ with a resolution of 4 cm^-1^. The X-ray (incident beam) used as a source consisted of radiation (λ = 1.5405 Å) with 30 kV power and 25 mA current. The adsorbents (i.e., before adsorption and after adsorption) were scanned at a scan rate of 0.02° min^-1^ within the 2*θ* range of 10° to 80°, after which the intensity (counts) was measured. SEM images were obtained at an electron acceleration voltage of 5 kV and magnifications of 300 and 600. The BET surface area was measured via the N_2_ adsorption‒desorption technique at liquid nitrogen temperature with a Nova 4000e (Quantachrome, USA). Before conducting the adsorption‒desorption tests, the samples were heated to 300 °C for 8 h to remove gas.

### 2.3. Adsorbent collection and preparation

**Fig 1:**
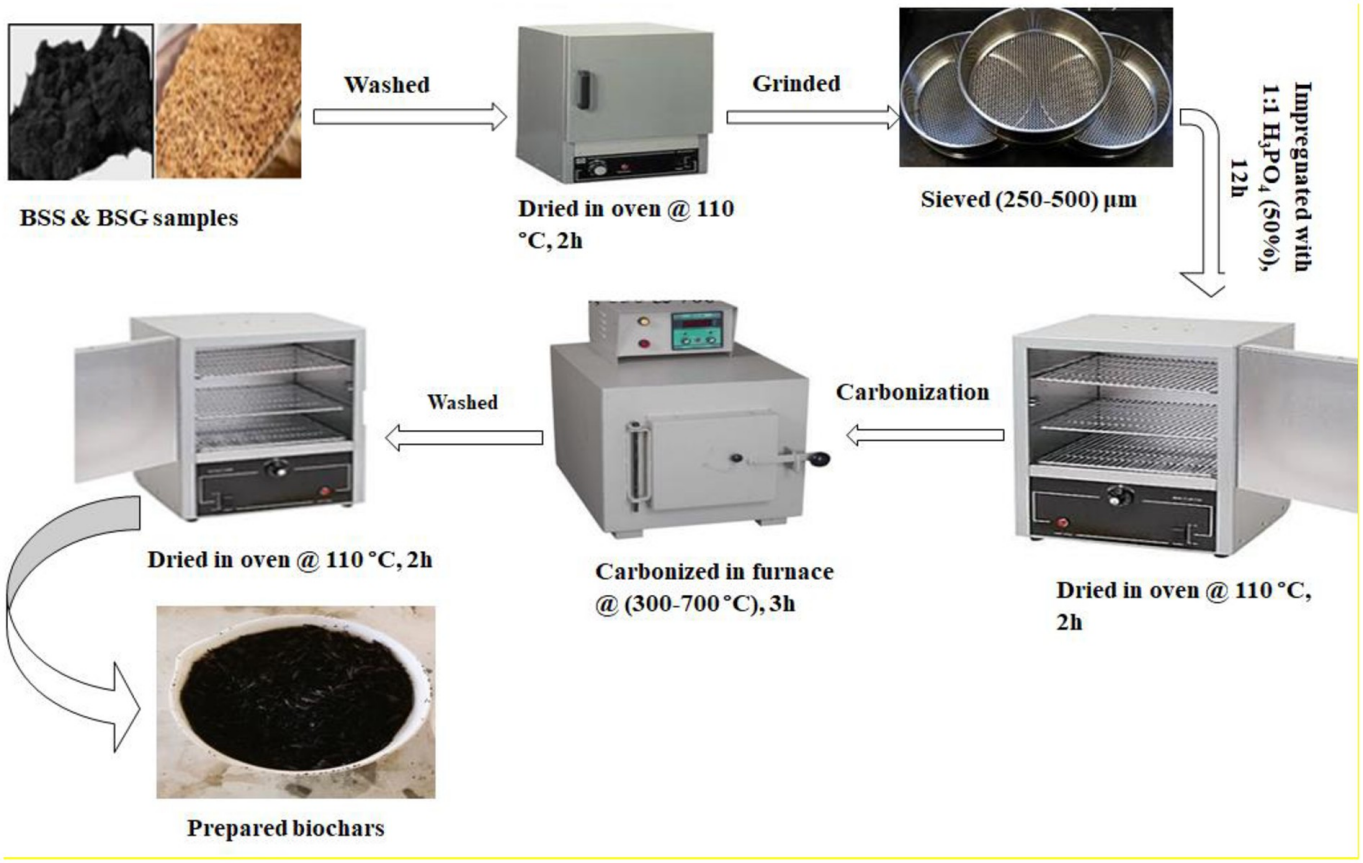
Schematic representation of the preparation of biochars from BSG, BSS and MIX.

Both the BSG and BSS samples were collected from Bedele Brewery Share Company and transported to the Analytical Chemistry Laboratory of Jimma University. The biomasses were washed thoroughly with distilled water to remove dirt and unwanted particles that had adhered to them. The samples were dried in an oven at 110°C for 2 h. Then, the dried samples were crushed and ground. The powder was sieved through a sieve with a particle size ranging from 250–500 μm. Powders of BSG, BSS and their mixture in a 1:1 ratio (MIX) were impregnated with a 1:1 phosphoric acid (50% w/w) activating agent for 12 h (Fig 1). The impregnated mixtures (adsorbent raw materials and activating agent) were dried in an oven at 110°C for 2 h. Then, the samples were carbonized in a muffle furnace at 300, 400, 500, 600, 650 and 700°C for 3 h under limited air. The resulting biochars were removed from the furnace and washed with distilled water several times [18, 19]. The biochar samples were subsequently dried in an oven at 110°C for 2 h, after which the products were collected and stored for further analysis. The adsorbent samples were labeled as untreated with acid and heat (UT-1), heat treated only at 600 °C (UT-2), acid treated only (AT-1), and acid impregnated followed by heat treatment at different temperatures, such as 300 °C (AT-2), 400 °C (AT-3), 500 °C (AT-4), 600 °C (AT-5), 650 °C (AT-6) and 700 °C (AT-7). For more information, see the supporting information (S1 Table).

### 2.4. Characterization of adsorbents

The moisture contents of the selected adsorbents (UT-1 and AT-5 of BSG and BSS) were determined following the methods of a previous study [20]. The ash content and volatile matter of the samples were determined via techniques applied in the literature [21]. The fixed carbon content of the samples was calculated by subtracting the summation of the percentage of all proximate analyses (moisture content, volatile matter content and ash content) from 100%. The point of zero charge (PZC) of BSG UT-1 and BSG AT-5 was studied at different background electrolyte concentrations of 0.01 M, 0.1 M and 1 M KNO_3_ solutions [22]. The BET surface area and pore size were determined via a Nova 4000e (Quantachrome, USA). The functional groups on the surface of the BSG biochar (before and after adsorption) were studied via FTIR. XRD is a powerful nondestructive technique that characterizes the presence of amorphous and crystalline phases in a given material through diffraction at an angle of 2*θ*. The morphological features of the prepared BSG biochar before and after adsorption of Cr(VI) were investigated via SEM (JCM-6000plus).

### 2.5. Batch adsorption studies

For screening of the untreated and acid-treated adsorbents at room temperature, the biochars at different experimental temperatures were tested with 20 mg/L Cr(VI), pH 2.5, and shaken for 24 h with a 2 g/L adsorbent. For further refinement of the adsorbents, screening was carried out for selected adsorbents at higher concentrations of Cr(VI), that is, 50 mg/L, pH 2.5, shaken for 24 h at a 2 g/L dose. Finally, the mixtures were filtered, and the filtrate was analyzed by UV‒Vis spectroscopy at 544 nm after treatment with the complexing agent 1,5-diphenylcabazide. All batch adsorption experiments were carried out at room temperature with agitation on a horizontal water bath shaker (GrantGLS400, England). The pH of the desired solution was adjusted by adding 0.1 M NaOH and 0.1 M HCl. Batch adsorption studies were carried out using 50 mL of 10 mg/L to 160 mg/L Cr(VI) solution and 0.1 to 6 g/L adsorbent in plastic jars at different contact times and solution pH values after agitation of the mixture with a horizontal shaker at 200 rpm.

After adsorption, the adsorbent was separated from the solution through filtration with Whatman No. 1 filter paper. The final Cr(VI) concentration was determined by adding 500 μL of 1 M H_2_SO_4_ and 500 μL of freshly prepared DPC solution (250 mg in 50 mL of acetone), allowing the mixture to stand for 10 min until purple-colored species formed; then, the concentration was analyzed via a UV‒Vis spectrophotometer at a wavelength of *λ* = 544 nm. Similarly, the initial concentration of the control for each experiment was analyzed via a UV‒Vis instrument. The constitution of tannery wastewater is usually complicated and involves multiple anions and cations. However, Cr(VI) exists as an anionic species over a wide pH range in aqueous environments [23]. Therefore, the effects of different anionic interferences on Cr(VI) adsorption were studied. Approximately 45 mL of a solution containing 20 mg/L Cr(VI) and the desired concentrations of the interfering ions (i.e., 2000 mg/L Cl^-^, 500 mg/L SO_4_^2-^, 250 mg/L NO_3_^-^, 100 mg/L PO_4_^3-^ and a mixture of them) were tested at the optimal contact time, dose, and pH. The extent of Cr(VI) removal efficiency and adsorption capacity were investigated under optimal conditions via Eqs (1) and (3), respectively. All of the experiments were performed in duplicate, and the mean value was reported. The removal efficiency of Cr(VI) is described by Eq (1) [24].

$$Removal\;efficiency\left(\%\right)=\frac{C_{0}-C_{t}}{C_{0}}\times 100$$ (1)

where C_o_ is the initial concentration (mg/L) and C_t_ is the concentration at a given time (mg/L).

The adsorption amount at one time (q_t_) and at equilibrium (q_e_) are determined via Eqs (2) and (3) [24].

$$q_{t}=\frac{(C_{0}-C_{t})V}{m}$$ (2)

$$q_{e}=\frac{\left(C_{0}-C_{e}\right)V}{m}$$ (3)

where V is the volume of adsorbate (L), m is the mass of adsorbent (g), q_e_ is the equilibrium adsorption capacity (mg/g) and q_t_ is the adsorption capacity over time (mg/g). Parameters that influence adsorption, such as pH, contact time, adsorbent dose and initial concentration of Cr(VI), were optimized and studied.

### 2.6. Data analysis

#### 2.6.1. Adsorption isotherm studies

Isotherms are used to predict the behavior of Cr(VI) adsorption on the surface of an adsorbent and to correlate equilibrium adsorption data. Several isotherm equations are available for analyzing experimental sorption equilibrium parameters. To predict the adsorption processes of Cr(VI) onto the adsorbents, experimental data were fitted to the Langmuir and Freundlich isotherm models. The Langmuir isotherm, which hints at homogeneity, describes the monolayer coverage of a sorbate on a sorbent surface with no mutual interactions on the sorbent surface [25]. The Freundlich model is used in the process of sorption on heterogeneous surfaces, i.e., energy heterogenic surfaces. Typically, on heterogeneous surfaces, the areas where adsorption occurs differ in terms of adsorption energy. Consequently, the places with the highest adsorption energy are covered first, and then the places with lower energy are covered [26]. The nonlinear forms of the Langmuir and Freundlich isotherms are given by Eqs (4) and (5), respectively.

$$q_{e}=\frac{q_{m}\;b_{L}C_{e}}{1+b_{L}C_{e}}$$ (4)

$$q_{e}=K_{F}C_{e}^{\frac{1}{n}}$$ (5)

where C_e_ is the concentration of the sorbate at equilibrium (mg/L), q_e_ is the amount of sorbate sorbed at equilibrium (mg/g), q_m_ is the maximum adsorption capacity (mg/g), b_L_ is the Langmuir equilibrium constant, and (L/mg) K_F_ ((mg^1−1/n^ L^1/n^)/g) and n are Freundlich adsorption constants related to the adsorption capacity and sorption intensity, respectively.

#### 2.6.2. Kinetic studies

The efficiency of the adsorbent was further studied by evaluating the kinetics of the adsorption process. Kinetics is the dynamic process of adsorption and describes the rate at which the adsorbent adsorbs Cr(VI). Among the kinetic models, the pseudo-first-order (PFO) and pseudo-second-order (PSO) kinetic models are the most well-known and familiar models for studying adsorption kinetics and quantifying the extent of uptake. The pseudo-first-order (PFO) model identifies adsorption on the basis of the adsorption capacity of solids. The nonlinear forms of PFO and PSO are given by Eqs (6) and (7), respectively [27].

$$q_{t}=q_{e}\left(1-e^{-k_{1}t}\right)$$ (6)

$$q_{t}=\frac{k_{2}q_{e}^{2}t}{1+k_{2}q_{e}t}$$ (7)

where q_e_ and q_t_ represent the amount of metal ions adsorbed at equilibrium and at a given time (mg/g), respectively; t represents time (min); k_1_ represents the PFO rate constant (min^-1^); and k_2_ represents the rate constant of PSO (g/mg/min).

The intraparticle diffusion model considers the diffusion of adsorbate molecules within the pores of biochar. This model suggests that the rate-determining step of adsorption is controlled by intraparticle diffusion and is described by Eq (8) [7].

$$q_{t}=k_{diff}t^{\frac{1}{2}}+c$$ (8)

where k_diff_ is the intraparticle diffusion rate constant (mg/(g min)^0.5^), t is time (min), c is a constant that reflects the boundary layer thickness (mg/g), and q_t_ is the amount adsorbed at time (mg/g).

#### 2.6.3. Statistical tests

To determine which model best fit the provided experimental data, a nonlinear chi-square (χ^2^) statistical test was also used in addition to the correlation coefficient (R^2^). A smaller χ^2^ indicates a greater degree of similarity between the modeled and experimental data, whereas a larger χ^2^ indicates more variation between the two sets of data. χ^2^ was computed via Eq (9) [28].

$$\chi^{2}=\sum \frac{{\left(q_{e}-q_{e,cal}\right)}^{2}}{q_{e,cal}}$$ (9)

where q_e,cal_ is the equilibrium adsorption capacity (mg/g) computed from the model and where q_e_ is the experimental equilibrium adsorption capacity (mg/g). The correlation coefficient (R^2^) was calculated as follows in Eq (10) [28]:

$$R^{2}=\frac{\sum {\left(q_{max}-q_{e}^{-}\right)}^{2}}{\sum {\left(q_{max}-q_{e}^{-}\right)}^{2}+\sum {{(q}_{max}-q_{e})}^{2}}$$ (10)

where q_max_ is the equilibrium capacity (mg/g) obtained from the isotherm model, q_e_ is the equilibrium capacity obtained from the experiment (mg/g) and q_e_ is the mean of q_e_ (mg/g).

### 2.7. Reusability of adsorbent

The number of adsorption cycles on the adsorbent was tested by maintaining the optimum adsorption conditions, such as the contact time, pH, initial concentration, and adsorbent dose (7 h, 2, 40 mg/L, and 2 g/L). The experiment was performed by carefully filtering the supernatant Cr(VI) solution and then leaving the adsorbent for some time to dry before each cycle after adsorption. After Cr(VI) adsorption, the adsorbent was treated with the NaOH desorption agent. A total of 2 g/L saturated adsorbent was added to a 50 mL plastic jar, and a 0.01 M NaOH solution (pH = 12) was added and shaken for the predetermined time. The adsorption and desorption cycles were independently determined until the adsorption efficiency of the adsorbents decreased significantly. The filtrate was analyzed with 500 μL of 1 M H_2_SO_4_ and 500 μL of DPC by double-beam UV‒visible spectroscopy at a wavelength of 544 nm. Finally, the percentage of desorption was calculated via Eq (11) [29].


$$Desorption\;ratio=\frac{Amount\;of\;metal\;ion\;desorbed}{Amount\;of\;metal\;ion\;adsorbed}\times 100$$ (11)

## 3. Results and discussion

### 3.1. Screening of adsorbents

Initial screening tests were conducted to identify the adsorbents that were highly efficient at removing Cr(VI). To achieve this goal, three distinct adsorbents, BSG, BSS, and MIX, were tested for their absorption of Cr(VI) at different carbonization temperatures and at room temperature. The efficiency of Cr(VI) removal was determined for each scenario, and the findings are documented in Table 1.

**Table 1:** Evaluation of the absorbent materials: Testing with concentrations of 20 mg/L and 50 mg/L Cr(VI) at pH 2.5, 2 g/L adsorbent, 24 h contact time, shaking at 200 rpm, and 25°C.

Adsorbent	Removal (%) initial Cr (VI) concentration (20 mg/L)			Removal (%) initial Cr (VI) concentration (50 mg/L)		
	BSS	BSG	MIX	BSS	BSG	MIX
**UT-1**	6.74 ± 0.20	26.58 ± 0.95	10.79 ± 0.01	3.31 ± 0.57	9.71 ± 0.75	8.35 ± 1.36
**AT-1**	53.42 ± 0.14	90.69 ± 0.78	72.55 ± 1.01	30.57 ± 8.68	29.89 ± 0.63	40.12 ± 0.75
**AT-2**	**88.73 ± 1.25**	**99.98 ± 0.02**	**97.45 ± 0.16**	77.60 ± 2.56	84.11 ± 0.29	75.64 ± 0.43
**AT-3**	72.50 ± 0.67	**97.99 ± 1.41**	87.20 ± 0.84	NT	79.35 ± 1.18	NT
**AT-4**	42.66 ± 4.00	**99.96 ± 0.01**	87.05 ± 11.14	NT	85.12 ± 2.29	NT
**AT-5**	23.02 ± 8.21	**99.96 ± 0.01**	46.52 ± 2.14	NT	**95.88 ± 0.51**	NT
**AT-6**	NT	NT	NT	NT	92.91 ± 0.13	NT
**AT-7**	NT	NT	NT	NT	87.53 ± 1.24	NT
**UT-2**	NT	NT	NT	NT	2.34 ± 1.40	NT

NT = Not tested because these experiments did not impact the choice of adsorbents.

As shown in Table 1, among the first screening (i.e., Cr(VI) at 20 mg/L) of eighteen (18) samples, BSG AT-2, BSS AT-2, MIX AT-2, BSG AT-3, BSG AT-4 and BSG AT-5 (99.98 ± 0.02%, 88.73 ± 1.25%, 97.45 ± 0.16%, 97.99 ± 1.41%, 99.96 ± 0.01%, and 99.96 ± 0.01%, respectively) had almost all the other high removal efficiencies. Therefore, further screening of these adsorbents was carried out by changing the initial concentration from 20 mg/L to 50 mg/L and keeping the other parameters constant. In addition, as the temperature of biochar preparation increased, the removal efficiency of Cr(VI) slightly decreased for BSG AT-6. When the temperature was further increased to 700°C, the Cr(VI) removal efficiency decreased. As presented in Table 1, among the adsorbents tested, acid treatment at 600°C effectively removed approximately 95.88 ± 0.51% of the 50 mg/L Cr(VI) from the aqueous solution when the initial Cr(VI) concentration increased from 20 to 50 mg/L for further refinement. Therefore, BSG AT-5 was selected as the optimal temperature for the synthesis of the biochar and was termed **BSG biochar**.

### 3.2. Characterization of the adsorbent

#### 3.2.1. Physicochemical characteristics

The physicochemical characteristics of UT-1 and AT-5 for BSG as well as for those from BSS are shown in Table 2.

**Table 2:** Results of the proximate analysis of the adsorbent samples.

Parameters	Biochar Samples			
	BSG UT-1	BSG AT-5	BSS UT-1	BSS AT-5
**Moisture content**	8.45 *±* 0.30	3.60 *±* 0.05	10.18 *±* 0.04	3.48 *±* 0.38
**Ash content**	4.27 *±* 0.15	4.93 *±* 0.25	41.22 *±* 0.54	40.25 *±* 0.14
**Volatile matter**	72.90 *±* 0.82	25.55 *±* 0.30	40.10 *±* 0.70	25.95 *±* 0.21
**Fixed carbon**	14.38 *±* 0.99	65.92 *±* 0.15	8.50 *±* 0.35	30.32 *±* 0.11

The efficiency of adsorption is greater when the moisture content is lower because water molecules can block the pores of an adsorbent, affecting the binding sites of the adsorbent. Hence, the adsorption effectiveness decreases as the moisture content of the adsorbent increases. BSG UT-1 had a moisture content of 8.45 ± 0.30%, ash content of 4.27 ± 0.15%, volatile matter content of 72.90 ± 0.82%, and fixed carbon content of 14.38 ± 0.99%. Comparable values were recorded for moisture content (7.03%), ash (1.68%), volatile matter (79.42%), and fixed carbon (11.87%) in an untreated BSG sourced from a brewery in southern Brazil [30]. The results indicated that the local brewing characteristics did not significantly impact the BSG variation in each brewery.

The quality of the adsorbent is determined by the ash percentage; a high-quality adsorbent is indicated by a low ash percentage [31]. Hence, the ash content found in BSG AT-5 (4.93 ± 0.25%) was less than the ash content in the other BSS samples (40.25 *±* 0.14%), suggesting that it is more suitable for Cr(VI) adsorption. When the BSG sample was carbonized, the ash content increased from 4.27 *±* 0.15% to 4.93 *±* 0.25%. A similar trend was reported for BSG pyrolysis, in which the ash content increased from 4.32 *±* 0.05% to 4.83 *±* 0.13% [32]. The volatile content of BSG UT-1 was 72.90 ± 0.82%, whereas that of BSG AT-5 was 25.55 ± 0.30%, indicating that the acid-treated BSG biochar had a greater adsorption capacity than did the untreated BSG. Fixed carbon is the remaining solid combustion residue after moisture, ash, and volatile matter are removed from the sample. Generally, an increase in the fixed carbon content of the adsorbent leads to a higher adsorption efficiency. Table 2 shows that the fixed carbon content of untreated BSG UT-1 was 14.38 ± 0.99%, whereas that of acid-treated BSG AT-5 increased to 65.92 ± 0.15%. BSG AT-5 can remove over 99.9% of Cr(VI), whereas BSG UT-1 can only remove up to 26.6% (Table 1), indicating that the adsorption ability of acid-treated BSG biochar was superior to that of untreated BSG.

**Fig 2:**
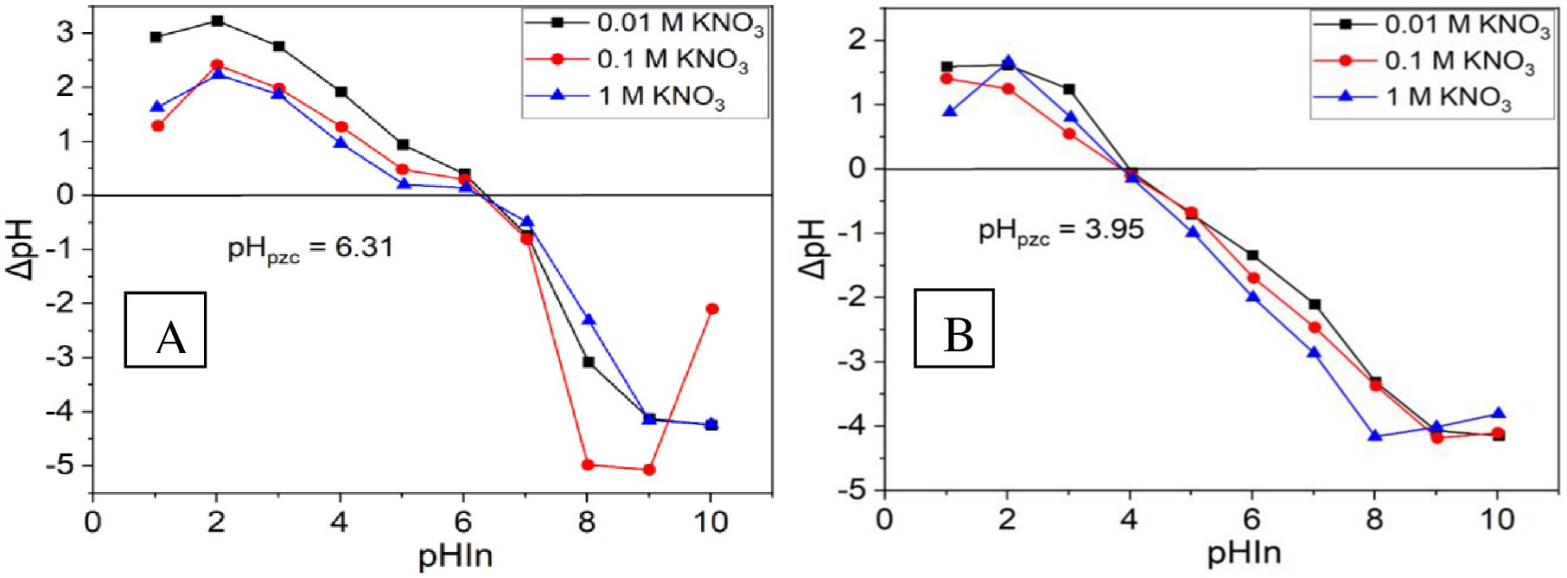
pH_PZC_ determination for (A) BSG UT-1 and (B) BSG AT-5.

BSG AT-5 biochar presented a greater specific surface area (889 m²/g) than did the BSG UT-1 adsorbent (523 m²/g). A similar biochar with a high surface area of 881 m²/g was recently reported from the base activation of solid waste distiller grains [16]. The BSG UT-1 adsorbent had a pore volume of 0.08 cm^3^/g, whereas the BSG AT-5 biochar had a greater value of 0.34 cm^3^/g. These findings suggest that activators enhance the porous nature of the adsorbent, leading to a higher adsorption capacity for the sorbent as the surface area and pore volume of the adsorbent increase. The adsorbents had pore radii of 0.18 nm (BSG UT-5) and 0.88 nm (BSG UT-1), suggesting that they were microporous. Fig 2 shows that the pH_PZC_ for BSG UT-1 was 6.31, whereas for BSG AT-5, it was 3.95. An analogous pattern was noted in both untreated sago hampas waste with a pH_PZC_ of 6.75 and acid-treated sago hampas waste with a pH_PZC_ of 3.04 [22]. In both instances, the pH_PZC_ decreased when the samples were subjected to H_3_PO_4_ treatment.

#### 3.2.2. Fourier transform infrared (FTIR) analysis

**Fig 3:**
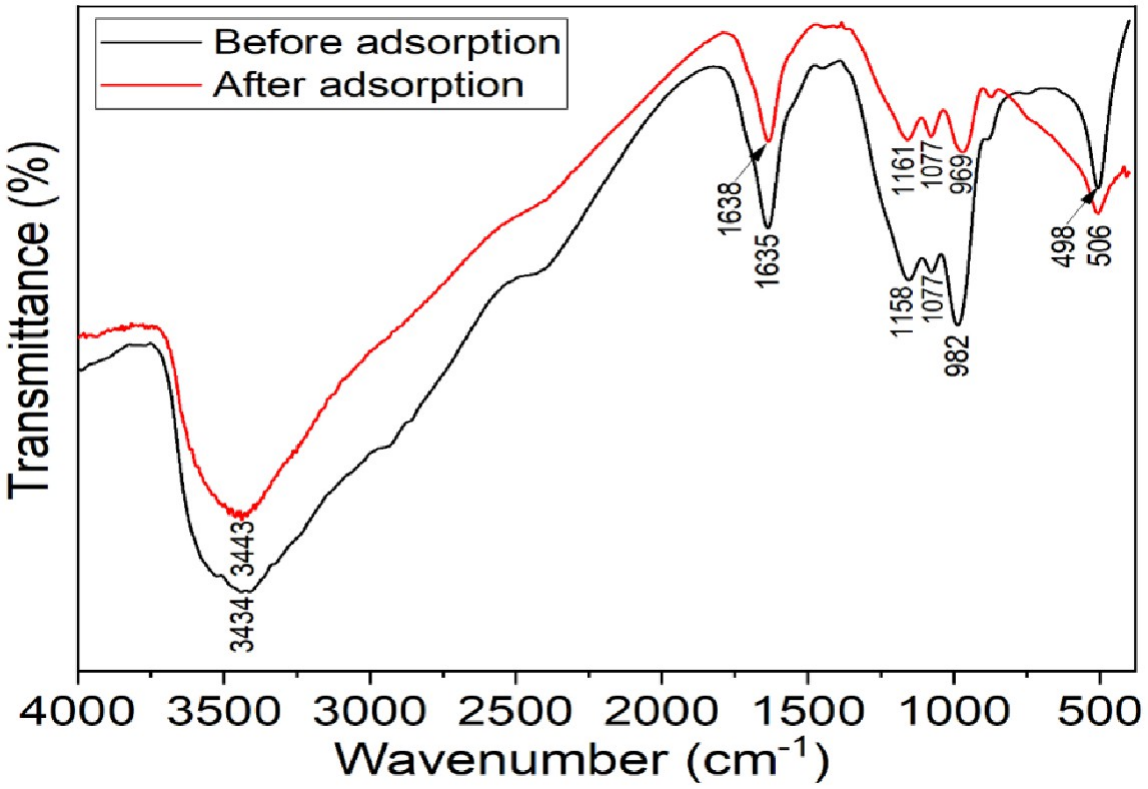
FTIR spectra of the BSG AT-5 biochar before and after adsorption.

FTIR analysis was conducted to determine the type of bonds present and to identify the various functional groups on the surface of the adsorbent. As shown in Fig 3, the broad, intense peak at approximately 3434 cm^-1^ before Cr(VJ) adsorption and 3443 cm^-1^ after Cr(VI) adsorption onto the BSG biochar indicate O-H and N-H stretching vibrations of polymeric compounds, such as lipids, proteins, cellulose, and lignin. The distinct peak observed at 1635 cm^-1^ before Cr(VI) adsorption and 1638 cm^-1^ after Cr(VI) adsorption corresponds to the occurrence of C = O stretching vibrations of amide bond linkages in the protein of the adsorbent. The same amide peak intensity was also observed by other scholars for the BSG adsorbent [33, 34].

The peaks between 950 cm^-1^ and 1200 cm^-1^ were attributed to C-O stretching and O-H bending in the carbonyl group [35]. The peak between 400 and 600 cm^-1^ indicates the existence of a metal‒oxygen connection [36], and the peak at 546 cm^-1^ corresponds to a Cr‒O bond [37]. Therefore, the weak peak at 506 cm^-1^ after the adsorption of BSG biochar indicates Cr–O bending vibrations. The spectra exhibited slight decreases in intensity and shifts in wavenumber after the adsorption of Cr(VI), suggesting the formation of bonds between Cr(VI) and the adsorbent surface [7].

#### 3.2.3. X-ray diffraction (XRD) analysis

**Fig 4:**
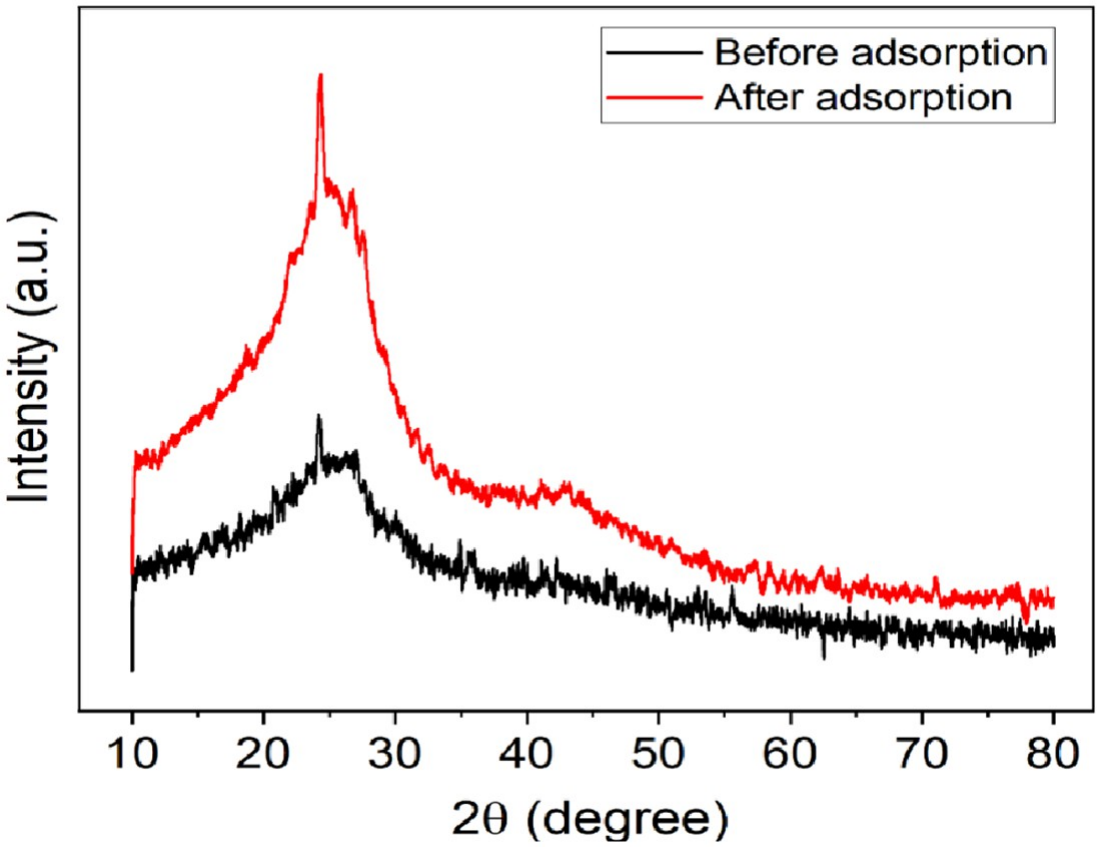
XRD patterns of the BSG AT-5 biochar before and after Cr(VI) loading.

XRD patterns were used to identify whether the samples contained crystalline or amorphous material. Fig 4 displays the XRD pattern of the BSG AT-5 biochar, which shows wide and less intense peaks, suggesting that the adsorbent is amorphous [16, 38]. On the other hand, the XRD pattern of the BSG AT-5 biochar after Cr(VI) adsorption shows medium intense peaks at 2θ values of approximately 23°. This peak indicated the presence of Cr(VI) on the surface of the adsorbent [39].

#### 3.2.4. Scanning electron microscopy (SEM) analysis

**Fig 5:**
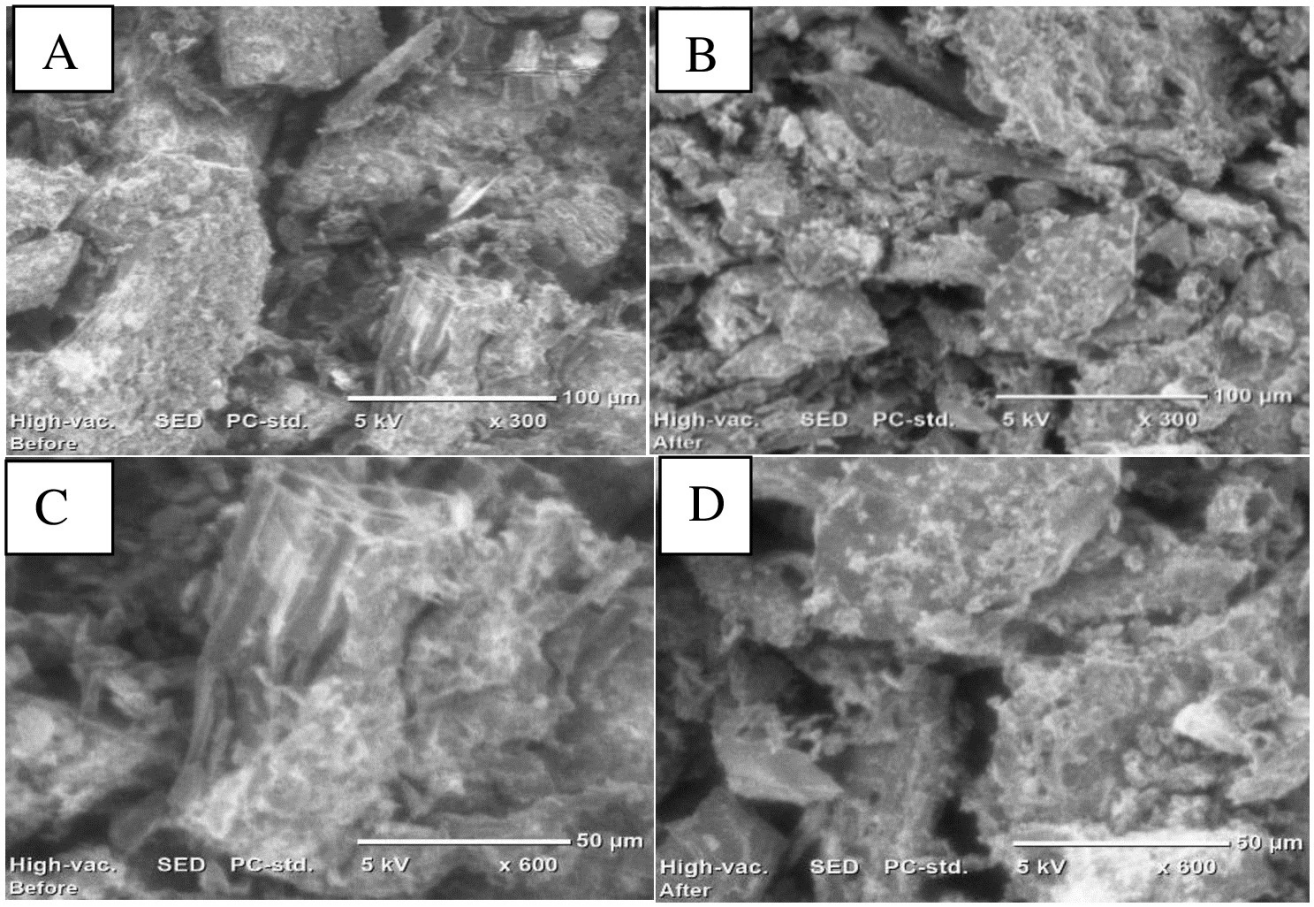
SEM images of BSG AT-5 biochar before adsorption (A & C) at 300 & 600× magnification and after adsorption (B & D) at 300 & 600× magnification.

SEM was used to examine the surface morphology of the BSG AT-5 biochar both before and after the adsorption of Cr(VI) at magnifications of 300 and 600x (Fig 5).

As shown in Fig 5A & 5C, the BSG AT-5 biochar surface exhibited an uneven texture with varying sizes and shapes of pores. According to Fig 5B & 5D, certain pores are blocked as a result of the adsorption of Cr(VI) onto the surface of the adsorbent, resulting in a smoother surface than the original rough surface. The attachment of Cr(VI) to the BSG biochar caused a layer of adsorbed substances to form on the surface, filling the surface cavities and pores and ultimately creating a smoother surface. The surface morphology of the Odaracha adsorbent showed a similar trend before and after Cr(VI) adsorption [40].

### 3.3. Effect of contact time

**Fig 6:**
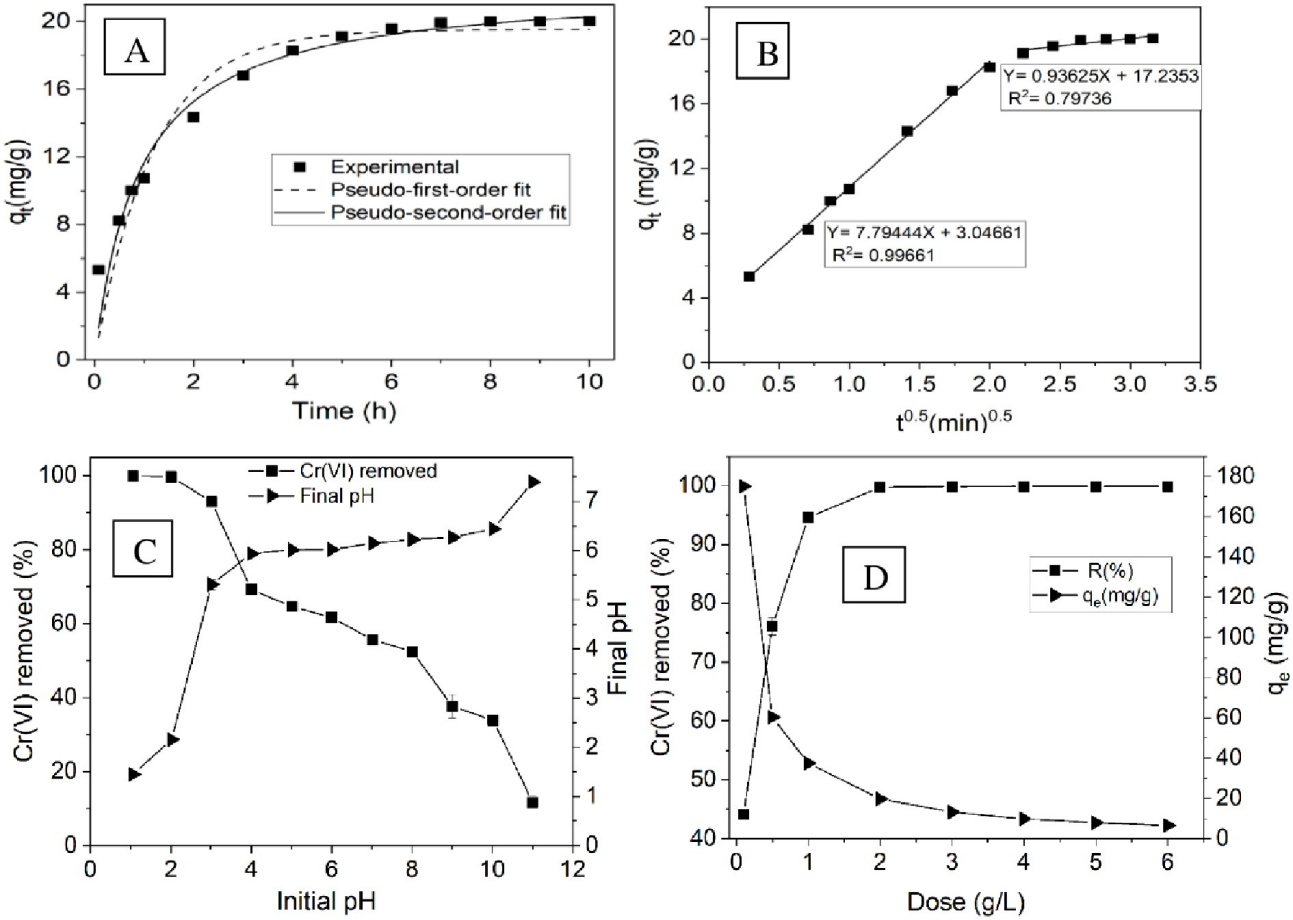
A) PFO and PSO plots, B) Intraparticle diffusion model plot, C) Effect of initial pH on Cr(VI) adsorption, D) Effect of adsorbent dose on Cr(VI) adsorption.

The impact of stirring duration on the removal of Cr(VI) was examined over a range of agitation times from 0.083–10 h. Initially, there were many empty active binding sites on the adsorbent, and a significant amount of Cr(VI) quickly attached to it at a high adsorption rate, as shown in Fig 6A. Over time, the number of available binding sites decreased, making it challenging for Cr(VI) to occupy the remaining surface sites due to repulsive forces between the Cr(VI) adsorbed on the solid surface and the liquid phase. The percentage of heavy metals removed increases as the contact time increases until reaching equilibrium [41]. The equilibrium time chosen for Cr(VI) adsorption was 7 h for further studies involving other parameters. In contrast to in situ nitrogenized BSG-activated carbon for Cr(VI) removal, BSG biochar removes Cr(VI) at a rate 2.3 times faster [14].

The nonlinear forms of the pseudo-first-order and pseudo-second-order kinetics were applied to the experimental data to assess the adsorption kinetics. This is because, in regard to determining the rate kinetic parameters, the nonlinear regression method is more suitable than the linear method is [27]. Both adsorption rate models were used to analyze the adsorption speed; the correlation coefficient (R^2^) and chi-square (χ^2^) were compared to identify the most suitable model. The data in Fig 6A and Table 3 indicate that the PSO algorithm has a higher R^2^ value of 0.9570 than does the PFO algorithm, which has an R^2^ value of 0.9265. Additionally, PSO had a lower χ^2^ value of 1.2912 than did PFO, with a χ^2^ value of 2.2083. The R^2^ value indicates how strong the correlation is between the predicted and actual values. An R^2^ value near 1 signifies an optimal model fit to the data. χ^2^ measures the disparity between the observed and predicted values, providing a gauge of the model’s goodness of fit. A smaller χ^2^ value suggests that the model fits the data well. The results of this study revealed that the PSO model was more suitable than the PFO model was, indicating the chemisorption of Cr(VI) onto the adsorbents [42].

**Table 3:** Parameters of the PFO and PSO kinetic models and isotherm parameters for Cr(VI) removal by BSG AT-5 biochar.

Parameters	Kinetic models		Isotherm models	
	PFO	PSO	Langmuir	Freundlich
**C**_**o**_ **(mg/L)**	40	40		
**q**_**e,exp**_ **(mg/g)**	20.005	20.005		
**q**_**e,cal**_ **(mg/g)**	19.5201	22.100		
**k**_**1**_ **(min**^**-1**^**)**	0.8498	-		
**k**_**2**_ **(g/(mg.min))**	-	0.0506		
**R** ^**2**^	0.9265	0.9570		
**χ** ^**2**^	2.2083	1.2912		
**q**_**cal**_ **(mg/g)**			31.87	-
**b (L/mg)**			90.33912	-
**R**_**L**_			6.97×10^−5^−1.10×10^−3^	-
**χ** ^**2**^			51.218	15.884
**K**_**F**_ **((mg**^**1−1/n**^ **L**^**1/n**^**) /g)**			-	23.413
**n**			-	7.565
**R** ^**2**^			0.6632	0.8956

To obtain a better understanding of the adsorption process and the factors affecting its speed, we studied the Weber‒Morris intraparticle diffusion model [43]. If intraparticle diffusion was the determining factor, the intraparticle graph would be a straight line passing through the origin. Nonetheless, if the data display multiple linear plots, the sorption process is governed by multiple processes. Fig 6B shows two separate linear regressions, indicating that adsorption is influenced by both film diffusion and intraparticle diffusion. Film diffusion occurred in the first stage of rapid growth, whereas intraparticle diffusion, identified as the rate-controlling step, occurred in the second phase [44]. Both the outer film and internal pore diffusion mechanisms played a role in the adsorption of Cr(VI) onto BSG biochar.

### 3.4. Effect of pH

The initial pH of the solution is a crucial parameter in most adsorption processes, as it can greatly impact both the extent and mechanism of adsorption. As shown in Fig 6C, the effectiveness of BSG AT-5 biochar in removing substances increased at lower pH values. As the pH increased from 2 to 11, the removal percentage decreased gradually from 99.70% to 11.70%. Hence, the most suitable pH was determined to be 2, similar to the pH levels commonly found in industrial waste, such as electroplating, chrome plating, and tannery effluent [45]. The findings concerning how pH affects adsorption could be explained by the way Cr(VI) behaves as an ion at various pH values. In a solution with water, Cr(VI) exists in various forms: chromic acid (H_2_CrO_4_), hydrogen chromate (HCrO_4_^−^), chromate (CrO_4_^2−^), and dichromate (Cr_2_O_7_^2−^). The pH determines the distribution of these species. H_2_CrO_4_, HCrO_4_^−^, and Cr_2_O_7_^2−^ are the three species of Cr(VI) found in aqueous solutions at lower pH values ranging from 1–6, whereas at higher pH values, the Cr(VI) species transition to CrO_4_^2−^ [23]. At lower pH values, HCrO_4_^−^ becomes the dominant species in the solution due to the high concentration of protons, leading to the adsorbent’s active sites being positively charged through protonation of functional groups such as amines and carboxyl groups [46].

The pH_PZC_ of the BSG biochar was determined to be 3.95. When the pH of the solution is lower than the pH_PZC_ (3.95), the surface of the BSG AT-5 biochar becomes positively charged, which helps in the adsorption of Cr(VI) ions. Therefore, at a lower pH, Cr(VI) ions are taken in through electrostatic attraction between the positively charged adsorbent and the negatively charged chromium species, particularly HCrO_4_^‐^ [47]. Nevertheless, if the pH exceeds 3.95, the surface of the BSG AT-5 biochar becomes negatively charged, which might lower the effectiveness of Cr(VI) ion adsorption [7]. Therefore, increased pH results in significant competition for adsorption sites on the adsorbent surface between OH^-^ ions and CrO_4_^2-^ species, reducing the adsorption efficiency [47].

### 3.5. Effect of adsorbent dose

The percentage of Cr(VI) removed rose from 44.13 to 99.74% as the amount of adsorbent used increased from 0.1 g/L to 2 g/L but plateaued after surpassing 2 g/L, as presented in Fig 6D. The removal efficiency (%) of adsorbents generally increased with increasing dose. The adsorption capacity decreased from 175.0 mg/g to 6.6 mg/g when the adsorbent dose increased from 0.1 g/L to 6 g/L. Studies have shown that the adsorption of Cr(VI) increases with increasing ACBSG amount, from 0.2 g/L to 4 g/L, in 25 mL of 10 mg/L Cr(VI). The highest adsorption of Cr(VI) was over 98% with 1 g/L adsorbent [14]. Both studies noted that the efficiency of Cr(VI) removal improved as the adsorbent dose increased until reaching an optimal point, with no further improvements observed beyond that point. It was anticipated that as the amount of adsorbent in the solution increased, the number of active sites for the metal ions would also increase [48]. In this study, the optimal dose for achieving a high removal efficiency was found to be 2 g/L.

### 3.6. Effect of initial Cr(VI) concentration

**Fig 7:**
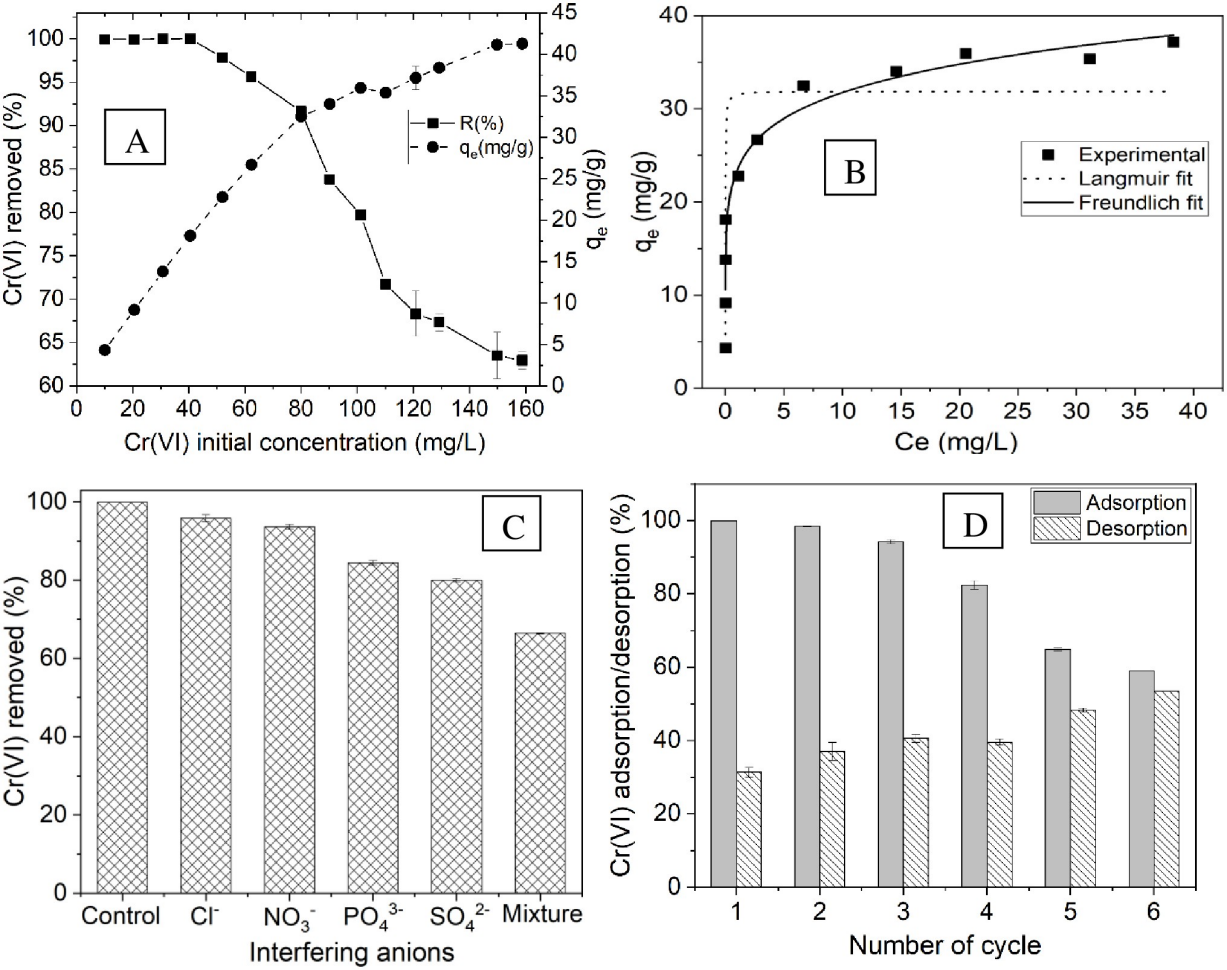
A) Effect of initial concentration on Cr(VI) adsorption, B) Langmuir and Freundlich isotherm model for the adsorption of Cr(VI) onto BSG AT-5 biochar, C) Effect of interfering ions on Cr(VI) removal, D) Adsorption‒desorption cycles for Cr(VI) removal by the BSG AT-5 biochar.

As the initial concentration of Cr(VI) increased, the removal efficiency decreased. As shown in Fig 7A, the percentage of Cr(VI) removed decreased from 99.96% to 62.94% as the initial Cr(VI) concentration increased from 10 mg/L to 160 mg/L. This decline occurred because, at lower concentrations, the adsorbent had adequate active sites for absorption. Nevertheless, when the concentration is increased, the surface area becomes inadequate for a significantly greater amount of Cr(VI) ions to be present in the solution [45]. At higher initial concentrations of Cr(VI), the adsorption capacity increased from 4.33 mg/g to 41.27 mg/g due to the elevated driving force for mass transfer when the initial Cr(VI) concentration increased from 10 to 160 mg/L. The impact of varying the initial Cr(VI) concentration (ranging from 25 to 100 mg/L) was evaluated in a study involving an acid-treated BSG adsorbent. As the concentration increased, the removal efficiency decreased from 78.6% to 62.3%, whereas the adsorption capacity increased from 3.93 mg/g to 12.42 mg/g [17]. Both studies noted that as the initial concentration of Cr(VI) increased, the removal efficiency decreased, and the adsorption capacity increased. This implies that both adsorbents reach a point of saturation at higher concentrations because of the limited number of active sites and exhibit a greater force for mass transfer at higher concentrations. As shown in Fig 7A, the removal of Cr(VI) increased linearly from 10 mg/L to 40 mg/L and then decreased gradually above 40 mg/L. As such, an initial concentration of 40 mg/L was deemed optimal for this research.

An adsorption isotherm can describe the adsorption of a solute on an adsorbent when equilibrium is achieved. Analysis of the experimental Cr(VI) adsorption data was conducted with the nonlinear Langmuir and Freundlich isotherm equations (Eqs (4) and (5)) at equilibrium. The experimental data were used to calculate the isotherm models and constants, with the findings displayed in Fig 7B and Table 3.

The most suitable isotherm model for describing the Cr(VI) adsorption process was determined by analyzing the correlation coefficient (R^2^) and chi-square (χ^2^) values. This study revealed that the Freundlich model had a higher R^2^ and lower *χ*^2^ than did the Langmuir model (Table 3). Hence, the Freundlich isotherm model was deemed most suitable for the experimental results of Cr(VI) adsorption onto BSG AT-5 biochar, indicating an uneven distribution of active sites on its surface. The calculated q_e_ value determined from the Langmuir plot was 31.87 mg/g, exceeding the q_max_ value documented in Table 4 in the literature. A greater calculated q_e_ value indicates a greater capacity for adsorption, possibly because of increased adsorption sites or stronger interactions between the adsorbent and Cr(VI). R_L_ is the crucial value derived from the Langmuir isotherm and is represented by a nondimensional equilibrium parameter, which is also referred to as the separation factor. The study revealed that the R_L_ varied from 0.0000697 to 0.0011. The value fell within the range 0 < R_L_ < 1, suggesting that Cr(VI) was effectively adsorbed on the BSG biochar [49].

**Table 4:** Comparison of the adsorption capacity of BSG biochar with that of previously reported adsorbents.

Adsorbents	q_max_ or the calculated q_e_ (mg/g)	Adsorption Conditions			Reference
		Equilibrium time (h)	pH	Dose (g/L)	
**Tella residue**	15.6	4	2	2.5	[7]
**Wheat bran**	0.942	1	3	20	[55]
**Pea seed shell**	8.5	1	3	2.5	[7]
**Paper mill sludge**	25.27		4	3.5	[52]
**Papaya peels**	7.16	3	1	10	[56]
**Vesicular basalt**	0.0792	9	2	50	[57]
**Commercial AC**	8.23	5	2	5	[58]
**BSG biochar**	31.87	7	2	2	**This study**

In the Freundlich equation, the parameter n represents the strength of the interaction between the adsorbent and solute, as well as the sorption capacity. When n is less than 1, adsorption weakens as the adsorbate binds, with favorable adsorption occurring at lower concentrations but becoming unfavorable at higher concentrations. When n equals 1, there is linear adsorption, resulting in constant uptake at all concentrations. A value of n greater than 1 indicates favorable adsorption across all concentrations, with uptake increasing as the concentration increases. When n is less than 1, adsorption is a chemical process; when n is equal to 1, adsorption is linear; and when n is greater than 1, adsorption is a physical process [50]. In this study, the calculated value of n was 7.565, indicating the favorability of the adsorption process across all concentration ranges and the involvement of a physisorption mechanism.

### 3.7. Effect of coexisting ions

To understand the effects of other ions present, tests were conducted with specific concentrations of interfering ions (i.e., 2000 mg/L Cl^-^, 500 mg/L SO_4_^2-^, 250 mg/L NO_3_^-^, 100 mg/L PO_4_^3-^ and a combination of all (mixture)) under optimal conditions of contact time, dosage, and pH. About 99.92% of the Cr(VI) ions were removed in the absence of any interfering ions. However, the removal efficiency decreased with the presence of various anions in the sequence Cl^-^ > NO_3_^-^ > PO_4_^3−^ > SO_4_^2−^ > mixture, achieving removal rates of 95.85%, 93.63%, 84.41%, 79.99%, and 66.41%, respectively, as shown in Fig 7C. This decrease in Cr(VI) removal efficiency was due to the competition between interfering ions and Cr(VI) for adsorption sites on the absorbent surface. The ranking of inhibitory strength was as follows: mixture > SO_4_^2−^ > PO_4_^3−^ > NO_3_^-^ > Cl^-^. An analogous pattern was observed in the extent of the suppression of Cr(VI) absorption by anions (SO_4_^2−^ > PO_4_^3−^ > NO_3_^-^) [51].

### 3.8. Reusability of the adsorbent

The increasing interest in the reusability of adsorbents is attributed to their potential for sustainable use. The Cr(VI) ion adsorption efficiency decreased from 99.96% to 59.04% over six cycles, as shown in Fig 7D. This decrease was caused by the active binding sites being filled with Cr(VI), leading to a decrease in the number of active sites with each cycle. Consequently, the efficiency of the adsorbent in removing Cr(VI) from the water solution decreased as the number of adsorption cycles increased [52].

As shown in Fig 7D, the desorption efficiency increased from 31.41% in the initial cycle to 53.50% in the sixth cycle. This surge can be linked to various factors, such as the diminished interactions between the adsorbate and adsorbent after repeated exposure to NaOH solution and the combined impact of desorption treatments across multiple cycles. Hence, the rate of desorption increases as the number of desorption cycles increases [52, 53]. The BSG AT-5 biochar showed greater reusability efficiency after six cycles, indicating that it can be repeatedly utilized for removing Cr(VI) from wastewater.

### 3.9. Mechanism of Cr(VI) adsorption

The initial pH of the solution is critical in adsorption processes, influencing both the extent and mechanism of adsorption. Under acidic conditions, Cr(VI) predominantly exists as HCrO₄⁻ and Cr₂O₇²⁻ ions, which are strongly attracted by the positively charged surface of biochar due to protonation. When the pH decreases below the pH_PZC_ of BSG AT-5 biochar (pH < 3.95), the biochar surface acquires a positive charge, increasing its electrostatic attraction to negatively charged Cr(VI) ions. Conversely, as the pH increases, the adsorption efficiency decreases sharply, suggesting that physiosorption is the primary mechanism. Similarly, Cr(VI) adsorption onto BSG AT-5 biochar aligns more closely with the Freundlich isotherm model than with the Langmuir model, indicating multilayer physisorption on its surface.

On the other hand, the FTIR spectra showed slight intensity decreases and wavenumber shifts after Cr(VI) adsorption (Fig 3), indicating chemical bonding between Cr(VI) and the adsorbent surface [7]. Moreover, the PSO model was more suitable than the PFO model, indicating the chemisorption of Cr(VI) onto the adsorbents [54]. Therefore, the adsorption of Cr(VI) onto BSG occurs through both physisorption and chemisorption.

### 3.10. Comparison of Cr(VI) adsorption onto various adsorbents

The proposed adsorption method for removing Cr(VI) was compared with those of other reported materials. Table 4 presents a comparison of the adsorption capacity, pH, adsorbent dosage, and equilibrium time of BSG AT-5 biochar for adsorbing Cr(VI) with those of other materials. The research findings showed that the BSG AT-5 biochar had better adsorption capability than the other adsorbents did. This could be attributed mostly to the carbon content, activation process, and pore development, along with the basic composition of the starting material [55].

### 3.11. Cost estimation of biochar production from BSG

The cost of producing biochar plays a significant role in the commercial use of biochar-based adsorption techniques for eliminating pollutants from water solutions. The total cost of producing biochar should consider different cost factors, such as the collection of local raw materials, processing needs, and pyrolysis conditions. The brewery’s spent grains (BSGs) are a type of brewing waste that can be easily found and collected locally at no cost. Phosphoric acid is also extremely affordable, with an average cost of US$ 1.7 per ton (https://www.alibaba.com) as of June 12, 2024 [59]. The expenses associated with washing, drying, and pyrolysis of the biomass were calculated via the current electricity tariff of 2.1240 Birr/kWh sourced from the official website of the Ethiopian Electric Utility on June 12, 2024 (http://www.ethiopianelectricutility.gov.et/contents/electricity-tariff) [60]. The cost of producing 1 ton of biochar from BSG was 208.5 US$ in the current study, as shown in S2 Table. The biochar production cost from BSG was lower (208.5 US $/ton) than the production cost of ferric-biochar composites (343.3 US $/ton) [61], indicating that biochar production from BSG was a cost-effective process. Therefore, biochar produced from brewery spent grain and phosphoric acid can serve as an affordable adsorbent for removing chromium from water.

## 4. Conclusion

In the present study, two brewery byproducts (BSG, BSS, and a combination of both) carbonized at different temperatures were tested to remove Cr(VI) from water. BSG AT-5 was chosen because of its superior removal efficiency (95.88%). It is an amorphous material with a high surface area (889 m²/g) and various functional groups that adsorb Cr(VI). The efficiency of Cr(VI) removal is affected by the adsorbent dose, contact time, pH, and initial solution concentration. Cr(VI) adsorption was better expressed with the PSO model than with the PFO model, indicating the chemisorption of Cr(VI) onto the adsorbents. The Freundlich isotherm model is better fitted than the Langmuir isotherm to the experimental equilibrium data, indicating that Cr(VI) was adsorbed onto the heterogeneous surface of the BSG biochar via physisorption. Therefore, we conclude that Cr(VI) adsorption onto BSG involves both physisorption and chemisorption.

The adsorption capacity estimated by the Langmuir isotherm model was 31.87 mg/g. The ability of BSG biochar to undergo six adsorption/desorption cycles suggests its potential for removing Cr(VI) from wastewater. Hence, BSG biochar could be beneficial for treating chromium-laden wastewater from industrial and other effluent sources before discharge into the aquatic environment under conditions that require further study. Additional research is recommended to examine how well the chosen adsorbent works with Cr(VI)-contaminated wastewater and its effectiveness in column adsorption procedures.

## Supporting information

S1 Table Screening of adsorbents. (DOCX)

S2 Table Cost estimation of biochar production via BSG. (DOCX)
