# Supplementary material for: Porous biochars derived from brewery waste for the treatment of Cr(VI)-contaminated water
Source: PLoS One. 2024 Nov 26;19(11):e0314522. doi: 10.1371/journal.pone.0314522 (PMC11594433; doi:10.1371/journal.pone.0314522)
Supplement: S1 Table — (DOCX) [file pone.0314522.s001.docx]

**ONE-D-24-13842**

**Porous biochars derived from brewery wastes for treatment of Cr(VI) contaminated water**

**PLOS ONE**

### **S1 Table.** **Screening of adsorbents**

| **Samples** | **Preparation at different experimental conditions** | **Nomenclatures** |
| --- | --- | --- |
| **BSG, BSS & MIX** | Untreated with acid and heat | UT-1 |
| **BSG** | Untreated with acid but heated at 600 °C | UT-2 |
| **BSG, BSS & MIX** | Acid Treated at room temperature | AT-1 |
| **BSG, BSS & MIX** | Acid Treated at 300 °C | AT-2 |
| **BSG, BSS & MIX** | Acid Treated at 400 °C | AT-3 |
| **BSG, BSS & MIX** | Acid Treated at 500 °C | AT-4 |
| **BSG, BSS & MIX** | Acid Treated at 600 °C | AT-5 |
| **BSG** | Acid Treated at 650 °C | AT-6 |
| **BSG** | Acid Treated at 700 °C | AT-7 |
