# Supplementary material for: Porous biochars derived from brewery waste for the treatment of Cr(VI)-contaminated water
Source: PLoS One. 2024 Nov 26;19(11):e0314522. doi: 10.1371/journal.pone.0314522 (PMC11594433; doi:10.1371/journal.pone.0314522)
Supplement: S2 Table — (DOCX) [file pone.0314522.s002.docx]

**ONE-D-24-13842**

**Porous biochars derived from brewery wastes for treatment of Cr(VI) contaminated water**

**PLOS ONE**

**S2 Table. Cost estimation of Biochar production using BSG**

| **Production process** | **Production cost**  **(US $/ ton)** |
| --- | --- |
| **Cost of BSG (since it was byproduct of brewery industry)** | 0 |
| **Transportation** | 11.7 |
| **Cost of washing BSG** | 35.4 |
| **Cost of drying** | 17.7 |
| **Phosphoric acid treatment** | 0.85 |
| **Pyrolysis of treated BSG** | 106.2 |
| **Cost of sample crushing** | 17.7 |
| **Overall cost** | 189.55 |
| **10% to overhead charge** | 18.96 |
| **Net cost for BSG biochar production** | 208.5 |
